# 2351. Systemic Influenza Infection Impairs Whole Blood Control of Mycobacteria in a Human Challenge Study

**DOI:** 10.1093/ofid/ofac492.158

**Published:** 2022-12-15

**Authors:** Claire Broderick, Oliver Powell, Samuel Nichols, Giselle D'Souza, Dominic Habgood-Coote, Zoe Gardener, Emma Bergstrom, Victoria Wright, Christopher W Woods, Christopher Chiu, Elizabeth Whittaker, Myrsini Kaforou, Sandra Newton, Michael Levin

**Affiliations:** Imperial College London, London, England, United Kingdom; Imperial College London, London, England, United Kingdom; Imperial College London, London, England, United Kingdom; Imperial College London, London, England, United Kingdom; Imperial College London, London, England, United Kingdom; Imperial College London, London, England, United Kingdom; Imperial College London, London, England, United Kingdom; Imperial College London, London, England, United Kingdom; Duke University Medical Center, Durham, North Carolina; Imperial College London, London, England, United Kingdom; Imperial College London, London, England, United Kingdom; Imperial College London, London, England, United Kingdom; Imperial College London, London, England, United Kingdom; Imperial College London, London, England, United Kingdom

## Abstract

**Background:**

Of the millions of people infected with *Mycobacterium tuberculosis* (Mtb) each year, only a small proportion will develop TB disease, with most immunologically containing or clearing Mtb. Factors influencing TB progression risk are incompletely understood. Co-infections, including influenza, have been proposed as a risk factor for TB progression via disruption of anti-Mtb immune responses. We employed a human influenza challenge study to investigate the effect of systemic influenza infection on host mycobacterial control.

**Methods:**

A whole blood (WB) mycobacterial growth inhibition assay was utilised to compare mycobacterial growth and anti-mycobacterial immune responses before and after influenza infection. Thirty adults were per-nasally inoculated with H3N2 influenza virus (Day [D] 0). Influenza PCR assay of D4 nasal swab confirmed infection. WB, collected pre- (D0, “pre-influenza”) and post-inoculation (D6, “post-influenza”), was infected with *Mycobacterium bovis* Bacille Calmette Guerin (BCG)-*lux*, incubated for 72 hours (h) and WB mycobacterial growth (growth ratio [GR]) measured. In parallel, BCG-*lux*-infected and uninfected blood aliquots were incubated for 0, 6, 24 and 72 h and measurements of cytokines (Meso Scale) and gene expression (RNA-Sequencing) undertaken. Comparisons between pre- and post-influenza infection blood samples were made (Fig. 1).

Blood was aliquoted into triplicate for each timepoint (0 hours [h], 72 h), diluted 1:1 with Gibco Roswell Park Memorial Institute 1640 Medium (RPMI), inoculated with a fixed quantity of BCG-lux and incubated at 37°C in a rocker-incubator. Previous experiments had confirmed and quantified the correlation between BCG-lux luminescence (measured in Relative Light Units [RLU] by a luminometer) and number of mycobacterial colony forming units. At 0 h and 72 h, the luminescence of each triplicate sample was measured in duplicate. The growth ratio (GR) was calculated by division of the median 72 h luminescence value by the median 0 h luminescence value. In parallel, BCG-lux inoculum or sterile PBS were added to additional whole blood aliquots (to give infected and uninfected aliquots), which were incubated at 37°C in a rocker-incubator. At timepoints 0 h, 6 h, 24 h, and 72 h, aliquots were removed from the incubator and centrifuged. Supernatants were frozen and stored for future cytokine analyses (quantification using Meso Scale Discovery [MSD] U-plex). Cell pellets were stabilised and stored for future RNA-sequencing and transcriptomic analysis. Figure created with BioRender.com.

**Results:**

In 22 influenza PCR-positive (+) subjects, median GR was significantly higher in post- (1.69) vs pre-influenza samples (1.03, p=0.0016) with no significant difference in the PCR-negative subjects (Fig. 2). Significant differences in BCG-*lux*-stimulated cytokine production were observed between post- and pre-influenza samples in PCR+ subjects (Fig. 3). Transcriptomic analysis identified significantly differentially expressed genes in the post- vs pre-influenza samples and differences between the groups over time, mapping to pathways related to TB susceptibility.

Complete BCG-lux growth ratio (GR) data were available for 28/30 participants. Comparisons of median GRs pre- and post-influenza infection for (A) 22 participants who were influenza PCR-positive on day 4 and (B) 6 participants who were influenza PCR-negative on day 4. Significance (paired analysis): ** p≤0.01; ns p>0.05. (C) Change in GR from pre-influenza baseline was calculated for each individual (GR-Post divided by GR-Pre, expressed as a percentage) and compared between the influenza PCR-positive and PCR-negative participants. Significance: ** p≤0.01.

Cytokine concentrations were measured in duplicate in supernatants from blood incubated with BCG-lux for 0 hours (h), 6 h, 24 h and 72 h, using Meso Scale Discovery U-plex. Results are available for a subset of 15 participants who had influenza infection confirmed by influenza PCR-positive assay on day 4 nasal swab. Comparisons of median concentrations between pre- and post-influenza samples and individual participants’ paired samples are shown for (A, B) Interleukin-1β (IL-1β) at 24 h; (C, D) Tumour necrosis factor-α (TNF-α) at 6 h; (E, F) Interleukin-10 (IL-10) at 0 h; (G, H) IL-10 at 24 h. Significance (paired analysis): **** p≤0.0001; ** p≤0.01.

**Conclusion:**

Systemic influenza infection reduces whole blood control of mycobacteria through modulation of anti-mycobacterial immune responses. Cytokine differences may contribute. These novel findings suggest that influenza may be a risk factor for TB disease and influenza vaccine could have a non-specific effect on TB immunity.

**Disclosures:**

**Christopher W. Woods, MD MPH**, Predigen, Inc: Co-founder **Christopher Chiu, BMBCh PhD**, GlaxoSmithKline: Grant/Research Support|Merck: Grant/Research Support|Reithera: Advisor/Consultant.

**Figure 1.**
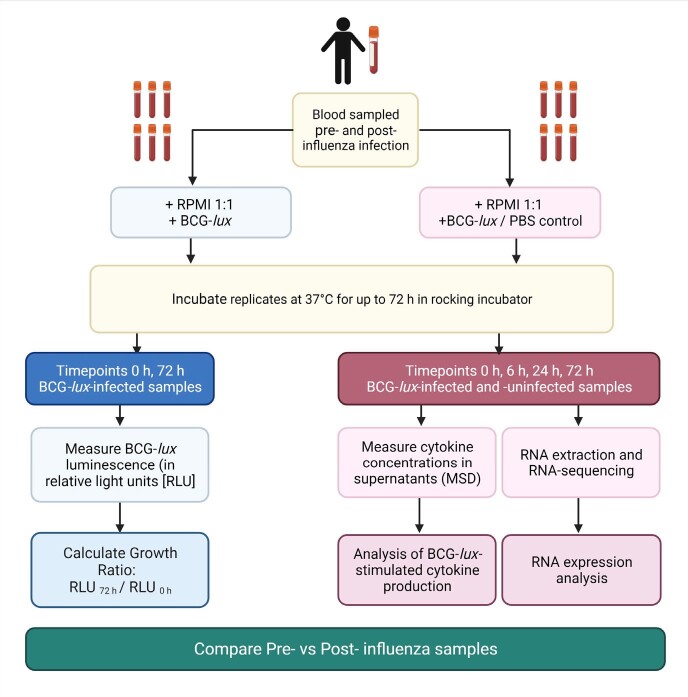
Overview of experimental workflow for study.

**Figure 2.**
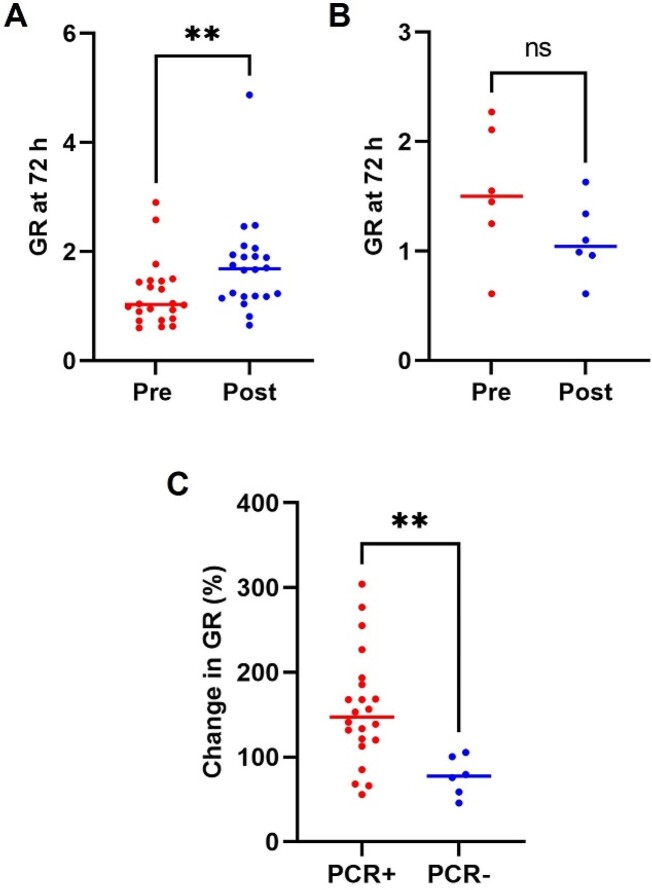
BCG-lux growth ratios at 72 h, before (pre) and after (post) H3N2 influenza infection.

**Figure 3.**
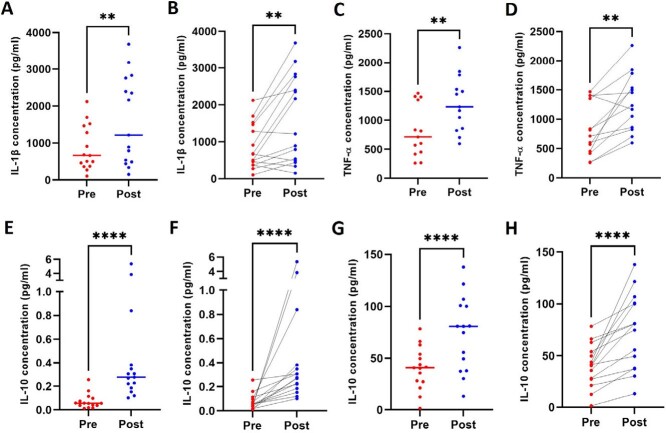
Cytokine production following whole blood culture with BCG-lux pre- and post-influenza infection.

